# Effect of feeding fermented mixture of cassava pulp and *Moringa oleifera* leaf meal on immune responses, antioxidative status, biochemistry indices, and intestinal ecology of broilers

**DOI:** 10.14202/vetworld.2020.392-399

**Published:** 2020-02-28

**Authors:** Sugiharto Sugiharto, Endang Widiastuti, Isroli Isroli, Turrini Yudiarti, Tri A. Sartono, Hanny I. Wahyuni

**Affiliations:** Department of Animal Science, Faculty of Animal and Agricultural Sciences, Diponegoro University, Semarang, Central Java, Indonesia

**Keywords:** antioxidant, broiler, fermented feed, health

## Abstract

**Aim:**

The study investigated the effect of feeding fermented mixture of cassava pulp and *Moringa oleifera* leaf meal (FCPMO) on the immune responses, antioxidative status, biochemical parameters, and intestinal ecology of broiler chickens.

**Materials and Methods:**

Four hundred Lohmann broiler chickens were distributed to four groups of diets including CONT (corn-soybean-based feed with no additive), BACI (corn-soybean-based diet supplemented with 0.1% zinc bacitracin), FERM (diet containing 20% FCPMO), and FERB (diet containing 20% FCPMO and added with 0.1% Bacillus subtilis). At days 4, 14, and 19, the chicks were vaccinated using commercial Newcastle disease-infectious bursal disease (ND-IBD), IBD, and ND vaccines, respectively. At day 35, blood was sampled and digesta was obtained from the ileum and caecum. Furthermore, the duodenal segment was obtained.

**Results:**

The BACI, FERM, and FERB groups had higher (p<0.05) serum superoxide dismutase activity than control. The malondialdehyde was lower (p=0.07) in BACI, FERM, and FERB than that in CONT. The BACI and FERM had lower (p<0.05) leukocytes and lymphocytes than CONT. The hemoglobin, erythrocytes, and hematocrit were lower (p<0.05) in BACI and FERM than those in CONT and FERB. Serum total triglyceride was lower (p<0.05) in FERM and FERB than that in CONT. The FERM and FERB had higher (p<0.05) albumin levels. Serum globulin level was lower (p<0.05) in FERB than that in BACI, but did not differ from that in CONT and FERM. The numbers of coliform, lactose-negative-enterobacteria and enterobacteria were lower (p<0.05) in FERB than that in other treatment groups. Crypt depth (CD) was higher (p<0.05) in FERM, while the villi height to CD ratio was lower (p<0.05) in FERM than that in CONT and FERB. The treatments showed no effect (p>0.05) on cecal volatile fatty acids production.

**Conclusion:**

Feeding FCPMO improved immune responses, antioxidative status, and physiological conditions, but had less effect on the intestinal ecology of broilers.

## Introduction

Feed efficiency is a critical factor for sustainable broiler enterprise worldwide. This is because feed accounts for more than 70% of the whole broiler production cost. Among the feed constituents, dietary energy sources (e.g., maize) occupy the major proportion of the broiler rations and thereby greatly determine the economics of the broiler industry. The continuous increase and the fluctuation in prices of the dietary energy sources have encouraged some farmers and broiler industries to exploit poor quality energy source feedstuffs. This practice may, however, result in less feed consumption, retarded growth rate, poor meat quality, and thus impaired overall economic performance [1]. Among the poor quality dietary energy sources, cassava pulp that is a by-product of tapioca production has widely been incorporated in broiler rations to reduce the proportion of maize partly. Yet, the extent of inclusion of cassava pulp is limited at a maximum of 8% from the total diets, as the greater extent of incorporation can compromise the growth and well-being of broilers [2]. To improve the nutritional qualities and hence enhance the dietary incorporation levels, fermentation has been applied on cassava pulp [3,4]. In most circumstances, urea may also be added in conjunction with the fermentation to elevate further the content of protein in cassava pulp [5,6]. Such urea supplementation can, however, harm the kidney and liver of the chickens particularly when the bioconversion of urea into microbial biomass protein is not completed during the fermentation [7,8]. Any alternative to further elevate the protein concentration of the cassava pulp based-fermented product is therefore beneficial.

The use of leaf meals as a source of protein in broiler diets has become a beneficial means to alleviate the cost of feed in poultry production. Apart from their high protein content, in general, leaf meals contain various anti-nutritional factors and toxins [9]. These latter properties may impair nutrient digestibility and utilization and also jeopardize the health of chickens. Indeed, fermentation has been documented to reduce the contents of anti-nutrition and toxins in the substrates [9,10]. In this regard, fermentation seems to be worthwhile for maximizing the utilization of dietary leaf meals by the chickens [8]. In this present study, *Moringa oleifera* leaf meal was mixed with cassava pulp before fermentation. Considering its high protein content [8,11], the *M. oleifera* leaf meal supplementation was expected to safely upgrade the protein content of cassava pulp, which was usually contributed by urea. A recent study revealed that feeding fermented feed was attributed to the improved immune responses, physiological conditions, and intestinal morphology and ecosystem of broiler chickens. Feeding such a diet has also been documented to improve the antioxidative status of broilers [9,10]. Besides being rich in protein, *M. oleifera* leaf meal has been documented to contain a myriad of phytochemical properties (e.g., antimicrobial and antioxidant agents) [11] that can promote the health of broiler chickens. In this study, the filamentous fungus *Chrysonilia crassa* was employed as the fermentation starter considering its fibrinolytic activity that can degrade the complex fiber into simple sugar [12]. Furthermore, the fungus exhibited probiotic properties [13], which may exert a beneficial impact on the health condition of broilers. Overall, it was expected that the combined effects of fermented feed, *M. oleifera* leaf meal and the fungus *C. crassa* would result in improved immune responses, antioxidative status, physiological conditions, and intestinal ecology of broiler chickens.

This study aimed to investigate the effect of feeding fermented mixture of cassava pulp and *M. oleifera* leaf meal (FCPMO) on the immune responses, antioxidative status, biochemistry indices, and intestinal ecology of broilers.

## Materials and Methods

### Ethical approval

The current experiment was approved by the Animal Ethics Committee of the Faculty of Animal and Agricultural Sciences, Diponegoro University, Indonesia and conducted in compliance with the standard procedures of raising livestock mentioned in the law of the Republic of Indonesia number 18, 2009, regarding animal husbandry and health.

### Production of fermented mixture of cassava pulp and *M. oleifera* leaf meal

The production of the FCPMO was preceded by the production of the fermentation starter. It began with the rejuvenation of the fungus *C. crassa* from the stored culture (preserved on potato dextrose agar [PDA] at 4°C). The rejuvenated fungi were then aerobically re-cultured on PDA for 48 h at 38°C. The spores of fungi were collected using 10 mL autoclaved distilled water. To produce the fermentation starter, 100 g of the used rice (purchased from the traditional market in Semarang) was cleaned and immersed in tap water for about 1 h. It was then steamed for 1 h and left on the tray until cool. The steamed used rice was subsequently inoculated with the fungal spore suspension (10 mL) as prepared above and aerobically incubated at room temperature for 48 h. The product from the latter process was sun-dried, milled, and eventually sieved before being used as a fermentation starter. Fungal colony enumeration (based on the plate count method) showed that the fermentation starter contained >1×10^8^ colony-forming unit/g of *C. crassa* colonies.

To produce the FCPMO, the dried cassava pulp containing ±12% of water content (purchased from the local supplier in Boyolali Regency, Central Java Province) was steamed for 1 h and permitted to cool thereafter. The *M. oleifera* leaf meal was prepared from the *M. oleifera* leaves collected from the gardens surrounding the university. The *M. oleifera* leaves were collected from March to May 2019. The *M. oleifera* leaves were spread, air-dried at room temperature and ground down into meal before use. For the production of the FCPMO, the steamed cassava pulp (60 g) was blended with *M. oleifera* leaf meal (35 g) and immediately inoculated with 5 g of fermentation starter (as prepared above). To obtain the moisture content of about 40%, the inoculated mixture was then poured with the sterilized distilled water (100 mL). The culture was aerobically incubated at about 25ºC (room temperature), and after 72 h, the FCPMO was sun-dried. After being taken for proximate analysis [14], the fermented product was stored at room temperature until use. The FCPMO contained 8.75% moisture, 17.6% crude protein, 4.41% crude fat, 9.01% crude fiber, and 10.1% ash, while cassava pulp contained 11.0% moisture, 2.24% crude protein, 0.91% crude fat, 31.8% crude fiber, and 3.65% ash. *M. oleifera* leaf meal contained 11.1% moisture, 29.9% crude protein, 5.38% crude fat, 13.2% crude fiber, and 12.6% ash.

### *In vivo* trial

Four hundred Lohmann broiler meat chickens were raised according to the commercial circumstances within the first 7 days. At day 8, the birds were allotted to four groups of diets, i.e. CONT (corn-soybean-based feed with no additive), BACI (corn-soybean-based diet administrated with 0.1% zinc bacitracin/in-feed antibiotic), FERM (diet with 20% of the FCPMO), and FERB (diet with 20% of the FCPMO and added with 0.1% probiotic *Bacillus subtilis*). Every group of the diet consisted of ten pens/replicates with ten birds per pen. The feeds were offered in mash form as a starter (Table-1) and finisher (Table-2) feed, and were offered *ad libitum* to all birds. Enzymes, antibiotics, antimolds, antiprotozoal, and anthelmintics were not incorporated in the diets. Through eye route, the chicks were vaccinated using the commercial Newcastle disease-infectious bursal disease (ND-IBD) vaccines on day 4. The chicks were also vaccinated using the commercial IBD vaccine on day 14 through drinking water. On day 19, the birds were vaccinated again with the commercial ND vaccine through drinking water. Along the period of trial, an opened-sided broiler house was used to raise the chicks. The litter/bedding was prepared from the rice husk. At the final of the experiment (day 35), ten birds from each diet (one bird per pen) were blood sampled (through wing veins) and subsequently slaughtered. The collected blood was placed either in vacutainers with ethylenediaminetetraacetic acid or vacutainers with no anticoagulant (to produce serum). To count the bacterial populations, digesta was obtained from the ileum of broilers. Furthermore, the cecal content was obtained for the determination of short-chain fatty acids (SCFA) production in the ceca. For the measurement of villi height and crypt depth (CD), around 2 cm of the duodenal segment was collected in 10% neutral formalin buffer solution.

**Table-1:** Constituents and chemical compositions of starter (days 8-21) diets.

Items (%, unless otherwise noted)	Dietary groups			

	CONT	BACI	FERM	FERB
Yellow maize	54.8	54.8	38.5	38.5
SBM	35.7	35.7	32.3	32.3
MBM	4.70	4.70	4.25	4.25
Soybean oil	1.55	1.55	1.75	1.75
FCPMO	-	-	20.0	20.0
DL-methionine, 990 g	0.30	0.30	0.30	0.30
L-Lysine, 780 g	0.20	0.20	0.20	0.20
Limestone	0.50	0.50	0.50	0.50
DCP	1.50	1.50	1.50	1.50
Premi×^1^	0.50	0.50	0.50	0.50
Salt	0.25	0.25	0.25	0.25
Calculated compositions				
ME^2^ (kcal/kg)	2900	2900	2900	2900
Crude protein	22.0	22.0	22.0	22.0
Crude fiber	5.60	5.60	6.30	6.30
Ca	1.10	1.10	1.10	1.10
P (available)	0.70	0.70	0.70	0.70
Lysine	1.20	1.20	1.20	1.20
Methionine	0.60	0.60	0.60	0.60

1 Premix contained (per kg of diet) of Vitamin A 7750 IU, Vitamin D3 1550 IU, Vitamin E 1.88 mg, Vitamin B1 1.25 mg, Vitamin B2 3.13 mg, Vitamin B6 1.88 mg, Vitamin B12 0.01 mg, Vitamin C 25 mg, folic acid 1.50 mg, Ca-d-pantothenate 7.5 mg, niacin 1.88 mg, biotin 0.13 mg, BHT 25 mg, Co 0.20 mg, Cu 4.35 mg, Fe 54 mg, I 0.45 mg, Mn 130 mg, Zn 86.5 mg, Se 0.25 mg, L-lysine 80 mg, choline chloride 500 mg, DL-methionine 900 mg, CaCO_3_ 641.5 mg, dicalcium phosphate 1500 mg.

2 Metabolizable energy was calculated on the basis of formula (Bolton, 1967) as follow: 40.81 {0.87 [crude protein+2.25 crude fat+nitrogen+free extract]+2.5}. CONT=Chicks received corn-soybean-based diet without additive, BACI=Chicks received corn-soybean-based diet supplemented with 0.1% zinc bacitracin, FERM=Chicks received diet containing 20% of the FCPMO, FERB=Chicks received diet containing 20% of the FCPMO and supplemented with 0.1% probiotic *B. subtilis*, SBM=Soybean meal, MBM=Meat bone meal, FCPMO=Fermented mixture of cassava pulp and *M. oleifera* leaf meal, DCP=Dicalcium phosphate, ME=Metabolizable energy, *B. subtilis=Bacillus subtilis*, *M. oleifera*=*Moringa oleifera*

**Table-2:** Constituents and chemical compositions of finisher (days 22-35) diets.

Items (%, unless otherwise noted)	Dietary groups			

	CONT	BACI	FERM	FERB
Yellow maize	58.5	58.5	42.4	42.4
SBM	32.7	32.7	28.8	28.8
MBM	2.35	2.35	2.25	2.25
Soybean oil	3.25	3.25	3.35	3.35
FCPMO	-	-	20.0	20.0
DL-methionine, 990 g	0.30	0.30	0.30	0.30
L-Lysine, 780 g	0.20	0.20	0.20	0.20
Limestone	0.50	0.50	0.50	0.50
DCP	1.50	1.50	1.50	1.50
Premix^1^	0.50	0.50	0.50	0.50
Salt	0.25	0.25	0.25	0.25
Calculated compositions				
ME^2^ (kcal/kg)	3060	3060	3060	3060
Crude protein	20.0	20.0	20.0	20.0
Crude fiber	5.60	5.60	6.30	6.30
Ca	1.10	1.10	1.10	1.10
P (available)	0.70	0.70	0.70	0.70
Lysine	1.20	1.20	1.20	1.20
Methionine	0.60	0.60	0.60	0.60

1 Premix contained (per kg of diet) of Vitamin A 7750 IU, Vitamin D3 1550 IU, Vitamin E 1.88 mg, Vitamin B1 1.25 mg, Vitamin B2 3.13 mg, Vitamin B6 1.88 mg, Vitamin B12 0.01 mg, Vitamin C 25 mg, folic acid 1.50 mg, Ca-d-pantothenate 7.5 mg, niacin 1.88 mg, biotin 0.13 mg, BHT 25 mg, Co 0.20 mg, Cu 4.35 mg, Fe 54 mg, I 0.45 mg, Mn 130 mg, Zn 86.5 mg, Se 0.25 mg, L-lysine 80 mg, choline chloride 500 mg, DL-methionine 900 mg, CaCO_3_ 641.5 mg, dicalcium phosphate 1500 mg.

2 Metabolizable energy was calculated on the basis of formula (Bolton, 1967) as follow: 40.81 {0.87 [crude protein+2.25 crude fat+nitrogen+free extract]+2.5}. CONT=Chicks received corn-soybean-based diet without additive, BACI=Chicks received corn-soybean-based diet supplemented with 0.1% zinc bacitracin, FERM=Chicks received diet containing 20% of the FCPMO, FERB=Chicks received diet containing 20% of the FCPMO and supplemented with 0.1% probiotic *B. subtilis*, SBM=Soybean meal, MBM=Meat bone meal, FCPMO=Fermented mixture of cassava pulp and *M. oleifera* leaf meal, DCP=Dicalcium phosphate, ME=Metabolizable energy. *B. subtilis=Bacillus subtilis*, *M. oleifera*=*Moringa oleifera*

The blood profile of chicks was assessed with a hematology analyzer (Prima Fully-auto Hematology Analyzer, PT. Prima Alkesindo Nusantara, Jakarta, Indonesia). The levels of antibody titers against the ND vaccine in the serum were measured according to the hemagglutination inhibition (HI) test, while the antibody titers against the IBD vaccine were based on the enzyme-linked immunosorbent assay method. The lipid profile (concentrations of total triglyceride, total cholesterol, high-density lipoprotein, as well as low-density lipoprotein) and concentrations of uric acid and creatinine in the serum were measured on the basis of the enzymatic colorimetric/color assays. The concentrations of alanine aminotransferase, aspartate aminotransferase, and total protein and albumin in the serum of chicks were measured according to the spectrophotometric/photometric assays. The data on globulin were generated from the serum total protein minus albumin. The aforementioned biochemical assays were run following the protocols from the manufacturer (DiaSys Diagnostic Systems GmbH, Holzheim, Germany). The levels of catalase, malondialdehyde (MDA), and serum superoxide dismutase (SOD) were spectrophotometrically determined with kits (Sigma-Aldrich, St. Louis, USA).

The histologic analysis was conducted using 5 µm of duodenal slice dyed with hematoxylin and eosin. The height of villus and depth of crypt were determined using an optical microscope fitted to a digital camera. The five measurements were used to obtain the mean of villi height and CD per chick. The selected bacteria numbers in the ileum of chicks were assigned based on Sugiharto *et al*. [15] with little changes. The coliform and lactose-negative enterobacteria were counted on MacConkey agar (Merck KGaA, Darmstadt, Germany) as red and colorless colonies after aerobic incubation (38°C, 24 h). The sum of coliform and lactose-negative enterobacteria was considered as *Enterobacteriaceae*. The same digesta sample was counted for the populations of lactic acid bacteria (LAB). The enumeration was conducted on de Man, Rogosa and Sharpe (MRS; Merck KGaA) agar following anaerobic incubation (38°C, 48 h). The levels of SCFA in the ceca of broilers were measured using gas chromatography based on the conditions explained by Sugiharto *et al*. [16].

### Statistical analysis

The data collected from the present trial were statistically analyzed with a one-way analysis of variance (SAS Inst. Inc., Cary, NC, USA). The latter analysis was applied to confirm whether there was a difference among the four treatment groups. When the remarkable (p<0.05) variation existed among the treatment groups, Duncan’s multiple range test was then conducted. This *post hoc* analysis was applied to determine the substantial difference among group means in the analysis of variance.

## Results and Discussion

Feeding fermented feed has usually been associated with improved immune functions in broiler chickens. Sugiharto and Ranjitkar [10] formerly revealed that fermented feed may increase the concentrations of the antibody as well as T cells of broiler chickens. The latter authors further suggested that the LAB contained in the fermented feed may induce the production of cytokines (by immune cells), which are capable of stimulating of broiler’s immune responses. Different from the latter report, data in our current study did not show any effect (p>0.05) of FCPMO on the IBDV and NDV titers (Table-3). With regard particularly to the NDV titer, feeding diets containing either zinc bacitracin, FCPMO, or FCPMO plus *B. subtilis* were associated with the more protected chickens from ND. Allan and Gough [17] formerly pointed out that HI titers below than 3 Log_2_ GMT are suggested as negative (not protected) for typical immunity toward ND. Data on the antioxidative status of broilers are listed in Table-3. Compared to CONT, the chicks in BACI, FERM, and FERB groups had higher (p<0.05) SOD activity. SOD has been noted as the first-line defense and most powerful antioxidants in the body of animals [18]. The higher SOD activity in the BACI, FERM, and FERB may, therefore, represent the less oxidative stress and hence less metabolic disorder in the respective birds when compared with the control. Corresponding to our data, the former study reported the increased SOD level in broiler chickens with zinc bacitracin supplementation [19]. In the previous report, we documented the antioxidant potentials of the filamentous fungus *C. crassa* [12,15]. In this respect, fermentation using the latter fungus may implicate in higher antioxidant potentials of the FCPMO resulting in the improved antioxidative status of broilers [9]. In addition to this, the antioxidant potential of *M. oleifera* leaf [11] seemed also to contribute to the improved antioxidant status of broiler chickens. With regard particularly to FERB, the birds from this group showed the highest SOD level in the current work. In addition to the antioxidative effects of fermented feed, *C. crassa* and *M. oleifera* leaf, the administration of probiotic *B. subtilis* may further corroborate the antioxidative defenses of FERB broilers as reflected by the SOD level. This was supported by the fact that feeding probiotic *B. subtilis* resulted in improved antioxidative status of broilers in the study of Abramowicz *et al*. [20]. In contrast to SOD levels, the serum level of MDA tended (p=0.07) to be lower in BACI, FERM, and FERB than that in CONT birds in the current study. MDA is one of the biomarkers of oxidative stress, at which the lower MDA level implies in the better antioxidative status of broilers. It was most likely that the antioxidative properties of fermented feed, the fungus *C. crassa*, *M. oleifera* leaf, as well as probiotic *B. subtilis* improved the antioxidant responses resulting in less oxidative damage in broilers.

**Table-3:** Immune responses and antioxidative status of broiler chickens fed treatment diets.

Items	Dietary groups				SE	p-value

	CONT	BACI	FERM	FERB		
IBDV titer (GMT)	11.8	11.9	11.8	11.6	8.57	0.59
NDV titer (Log_2_ GMT)	2.80	3.40	3.50	3.40	0.58	0.82
Catalase (ng/mL)	321	463	290	289	68.9	0.23
SOD (U/mL)	25.7^c^	28.7^b^	29.8^b^	31.5^a^	0.45	<0.01
MDA (nmol/mL)	2.57	2.12	2.10	1.35	0.33	0.07

a,b,c Means with various letters within the similar row indicate substantial difference. CONT=Chicks received corn-soybean-based diet without additive, BACI=Chicks received corn-soybean-based diet supplemented with 0.1% zinc bacitracin, FERM=Chicks received diet containing 20% of the FCPMO, FERB=Chicks received diet containing 20% of the FCPMO and supplemented with 0.1% probiotic *B. subtilis*, SE=Standard error, IBDV=Infectious bursal disease vaccine, NDV=Newcastle disease vaccine, GMT=geometric mean titer, SOD=Serum superoxide dismutase, MDA=Malondialdehyde, *B. subtilis=Bacillus subtilis*

Data on the blood profile of broilers are provided in Table-4. It was clear that chickens in the BACI and FERM groups had lower (p<0.05) leukocytes and lymphocytes numbers as compared particularly to the chickens in CONT group. A former study by Hidanah *et al*. [21] noticed the increased leukocytes and lymphocytes numbers following *Mycoplasma gallisepticum* infection. Similarly, Akhtar *et al*. [22] reported that infection with mixed *Eimeria* species increased the numbers of leukocytes and lymphocytes in broiler chickens. For these reasons, we may infer that the lower counts of leukocytes and lymphocytes in BACI and FERM birds seemed to be associated with the lower potential of infections in these respective birds. This seemed reasonable given that zinc bacitracin and fermented feed possessed the antimicrobial properties, which are possible to control the invading pathogenic microorganisms [10]. It has generally been known that infections may be linked to the increased metabolic rate in animals due to the increased need for maintenance (recovery) energy [23]. In this study, the hemoglobin, erythrocytes, and hematocrit values were lower (p<0.05) in BACI and FERM than that in CONT and FERB chickens. Due to the role of erythrocytes in transporting oxygen, and thus supporting the energy-generating metabolism, the higher levels of hemoglobin, erythrocytes, and hematocrit in the CONT and FERB chicks may, therefore, be associated with the higher potential of infections in these chickens. Nonetheless, the latter inference should be taken with care, as the values of hemoglobin and erythrocytes found in this current study were still in the normal ranges. Talebi *et al*. [24] suggested that healthy broilers have around 2.84×10^6^/µL erythrocytes and 13.94 g/dL hemoglobin.

**Table-4:** Complete blood counts of broiler chickens fed treatment diets.

Items	Dietary groups				SE	p-value

	CONT	BACI	FERM	FERB		
Leukocytes (10^3^μL)	69.8^a^	59.2^b^	58.9^b^	65.9^a,b^	2.61	0.01
Heterophils (10^3^μL)	2.22	2.15	2.45	2.30	0.40	0.95
Lymphocytes (10^3^μL)	67.6^a^	57.0^b,c^	56.4^c^	63.8^a,b^	2.46	0.01
Thrombocytes (10^3^μL)	12.3	10.4	10.3	10.7	1.64	0.79
Hemoglobin (g/dL)	9.61^a^	8.05^b^	7.45^b^	9.56^a^	0.43	<0.01
Erythrocytes (10^6^μL)	2.78^a^	2.36^b^	2.16^b^	2.76^a^	0.11	<0.01
Hematocrit (%)	34.9^a^	29.3^b^	26.7^b^	34.6^a^	1.53	<0.01
MCV (fL)	127	125	124	126	0.98	0.25
MCH (pg)	34.1^a^	34.1^a^	31.3^b^	34.6^a^	0.69	<0.01
MCHC (g/dL)	27.1	27.5	27.5	27.6	0.51	0.90

a,b Means with various letters within the similar row indicate substantial difference. CONT=Chicks received corn-soybean-based diet without additive, BACI=Chicks received corn-soybean-based diet supplemented with 0.1% zinc bacitracin, FERM=Chicks received diet containing 20% of the FCPMO, FERB=Chicks received diet containing 20% of the FCPMO and supplemented with 0.1% probiotic *B. subtilis*, SE=Standard error, MCV=Mean corpuscular volume, MCH=Mean corpuscular hemoglobin, MCHC=Mean corpuscular hemoglobin concentration, *B. subtilis=Bacillus subtilis*

Data on the serum biochemical parameters of broiler chickens are presented in Table-5. The level of serum total triglyceride was lower (p<0.05) in FERM and FERB than that in CONT broilers. Similar to our findings, Yamamoto *et al*. [25] revealed that feeding Koji-feed (fermented distillery by-product) resulted in decreased concentration of plasma triglyceride in broilers. It seemed that fermented feed enhanced the hydrolysis of triglycerides as well as fatty acid β-oxidation leading to reduced triglyceride concentrations in the circulatory system of broilers [10]. It was observed in this study that the FERM and FERB chicks had a higher (p<0.05) level of albumin in the serum. Besides helping in maintaining the metabolic balances, albumin has been suggested to contribute to tissue protein synthesis in broiler chickens. In this study, the higher level of albumin in FERM and FERB chicks was inversely associated with the level of triglycerides in the serum of the respective birds. The latter condition may suggest that feeding fermented feed shifted the metabolic process from fat synthesis to fatty acid β-oxidation and tissue protein synthesis. In agreement with our inference, Vieira *et al*. [26] reported the inverse relationship between serum albumin and triglycerides levels in broilers fed mango waste meal at 42 days of age. However, our inference must be taken with care as the increased albumin level was not capable of increasing the total protein in the serum of the respective birds. The same condition was actually found in the study of Vieira *et al*. [26], in which the albumin level was not in parallel with the total protein levels in the serum of broilers. Our present finding showed that serum globulin level was lower (p<0.05) in FERB than that in BACI, but did not differ from that in CONT and FERM broilers. In broilers, the globulin level is generally elevated in response to potential infections [27]. In this study, the intestinal populations of coliform, lactose-negative-enterobacteria, as well as enterobacteria were lower in FERB than that in other treatment birds. The lower intestinal pathogenic bacteria load could, therefore, be attributed to the less immune inducement and thus lower serum globulin level. In line with our inference, Manafi *et al*. [28] noticed that the lower loads of coliforms, *Salmonella*, and *Escherichia coli* in the intestine of broilers were associated with the lower level of serum globulin of broilers.

**Table-5:** Serum biochemical parameters of broiler chickens fed treatment diets.

Items	Dietary groups				SE	p-value

	CONT	BACI	FERM	FERB		
Total cholesterol (mg/dL)	115	108	95.3	101	6.44	0.16
HDL (mg/dL)	93.9	89.4	86.2	85.2	5.96	0.73
LDL (mg/dL)	13.3	10.8	11.4	8.52	4.13	0.88
Total triglyceride (mg/dL)	50.0^a^	44.8^a,b^	34.4^b^	35.2^b^	3.51	0.01
AST (U/L)	276	263	243	244	11.7	0.16
ALT (U/L)	1.40	1.49	1.54	1.46	0.41	0.99
Albumin (g/dL)	1.16^b^	1.17^b^	1.40^a^	1.29^a^	0.04	<0.01
Total protein (g/dL)	2.15^b^	2.35^a,b^	2.47^a^	2.21^b^	0.08	0.03
Globulin (g/dL)	0.99^a,b^	1.18^a^	1.07^a,b^	0.92^b^	0.07	0.03
Uric acid (mg/dL)	4.08	3.66	4.22	3.17	0.32	0.07
Creatinine (mg/dL)	0.01	0.03	0.03	0.02	0.01	0.52

a,b Means with various letters within the similar row indicate substantial difference. CONT=Chicks received corn-soybean-based diet without additive, BACI=Chicks received corn-soybean-based diet supplemented with 0.1% zinc bacitracin, FERM=Chicks received diet containing 20% of the FCPMO, FERB=Chicks received diet containing 20% of the FCPMO and supplemented with 0.1% probiotic *B. subtilis*, SE=Standard error, HDL=High-density lipoprotein cholesterol, LDL=Low-density lipoprotein cholesterol, AST=Aspartate aminotransferase, ALT=Alanine aminotransferase. *B. subtilis=Bacillus subtilis*

Data on the selected ileal bacterial counts of broilers are presented in Table-6. As previously mentioned, the numbers of coliform, lactose-negative-enterobacteria, and enterobacteria were lower (p<0.05) in FERB than that in other dietary groups. It seemed that the probiotic activities of *B. subtilis* played a substantial role in reducing the numbers of the potential pathogenic bacteria in the ileal digesta of broilers in the current work. The same effect of probiotic *B. subtilis* in reducing the pathogenic bacterial load in broiler intestine has also formerly been reported by Manafi *et al*. [28]. The present finding, however, partly differed from Manafi *et al*. [28], as the treatment with antibiotic zinc bacitracin did not reduce the numbers of pathogenic bacteria in the ileum of broiler in the present work. Recently, Sugiharto and Ranjitkar [10] suggested that fermented feed may be a tool to reduce the pathogenic bacteria in the intestine of broilers. However, fermented feed did not markedly influence the number of intestinal bacteria of broilers in the present study. The differences in the nature of raw feed ingredients, microorganisms used as a fermentation starter and the conditions of the experiment may account for the discrepancy above.

In comparison with other groups, the crypt was deeper (p<0.05) in FERM group in the present study, while the villi height was not significantly different across the dietary groups (Table-7). The corresponding results were actually revealed by Naji *et al*. [29], in which feeding fermented feed increased CD in the whole intestinal segments (duodenum, jejunum, and ileum) of broiler chickens. In contrast to our data, the increased CD in the latter study did not lower the ratio of villus height to CD (VH/CD) in the intestine of the respective broilers. In most circumstances, the higher VH/CD may indicate the better digestion and absorption capacity of the small intestine of broilers. The lower VH/CD in the duodenum of broiler in the present study may, therefore, reduce the absorptive capacity of chickens. The definite reason for the deeper crypt and hence lower VH/CD in FERM birds remains unknown, but the higher fiber content in the FERM diet seemed to be responsible. A recent study by Saadatmand *et al*. [30] showed that dietary fiber (rice hull) was attributed to the lower VH/CD in the jejunum and ileum resulting in weak absorptive capacity and impaired growth performance of broiler chickens. With regard to FERB birds, the detrimental effect of dietary fiber on the intestinal morphology seemed to be compensated by the presence of probiotic *B. subtilis*, which has been documented to increase the VH, decrease CD, and thus increase VH/CD [28].

**Table-6:** Ileal bacterial populations of broiler chickens fed treatment diets.

Items (log CFU/g)	Dietary groups				SE	p-value

	CONT	BACI	FERM	FERB		
LAB	11.4	11.3	11.4	11.2	0.10	0.24
Coliform	9.30^a^	9.41^a^	8.82^a^	8.09^b^	0.19	<0.01
Lactose- negative - enterobacteria	9.01^a^	8.99^a^	9.25^a^	8.36^b^	0.19	0.02
Enterobacteria	9.54^a^	9.59^a^	9.42^a^	8.72^b^	0.17	<0.01

a,b Means with various letters within the similar row indicate substantial difference. CONT=Chicks received corn-soybean-based diet without additive, BACI=Chicks received corn-soybean-based diet supplemented with 0.1% zinc bacitracin, FERM=Chicks received diet containing 20% of the FCPMO, FERB=Chicks received diet containing 20% of the FCPMO and supplemented with 0.1% probiotic *B. subtilis*, SE=Standard error, LAB=Lactic acid bacteria, CFU=Colony-forming unit, *B. subtilis=Bacillus subtilis*

**Table-7:** Duodenal morphology of broiler chickens fed treatment diets.

Items	Dietary groups				SE	p-value
	CONT	BACI	FERM	FERB		
Villi height (μm)	1355	1352	1476	1491	71.5	0.36
Crypt depth (μm)	211^b^	252^b^	368^a^	254^b^	27.1	<0.01
VH/CD	6.60^a^	5.54^a,b^	4.54^b^	6.09^a^	0.49	0.03

a,b Means with various letters within the similar row indicate substantial difference. CONT=Chicks received corn-soybean-based diet without additive, BACI=Chicks received corn-soybean-based diet supplemented with 0.1% zinc bacitracin, FERM=Chicks received diet containing 20% of the FCPMO, FERB=Chicks received diet containing 20% of the FCPMO and supplemented with 0.1% probiotic *B. subtilis*, SE=Standard error, VH/CD=Villi height to crypt depth ratio, *B. subtilis=Bacillus subtilis*

Data in our current study (Table-8) showed no substantial effect of treatments on the production of SCFA in the cecal digesta of broilers. In accordance with this finding, Sharma *et al*. [27] showed that feeding crimped kernel maize silage did not affect the production of SCFA in the ileum and cecum of broilers. In contrast to both studies, our previous work revealed that feeding *Acremonium charticola*-fermented cassava pulp resulted in greater production of butyric acid in the cecal digesta of broiler chickens [31]. Possibly, the different nature and nutritional qualities of the fermented products, the composition of the complete rations and the conditions of the studies were account for the divergent findings above.

**Table-8:** Short chains fatty acids concentrations in caecum of broiler chickens fed treatment diets.

Items (mmol/kg)	Dietary groups				SE	p-value

	CONT	BACI	FERM	FERB		
Acetic acid	155	112	131	193	33.4	0.37
Propionic acid	44.8	34.3	43.6	71.0	12.4	0.20
Butyric acid	46.5	36.9	40.5	67.6	11.6	0.26

CONT=Chicks received corn-soybean-based diet without additive, BACI=Chicks received corn-soybean-based diet supplemented with 0.1% zinc bacitracin, FERM=Chicks received diet containing 20% of the FCPMO, FERB=Chicks received diet containing 20% of the FCPMO and supplemented with 0.1% probiotic *B. subtilis*, SE=Standard error, *B. subtilis=Bacillus subtilis*

## Conclusion

Feeding FCPMO improved immune responses, antioxidative status, and physiological conditions, but had minimum impact on the intestinal ecology of broilers.

## Authors’ Contributions

SS designed, conducted the experiment, and prepared the manuscript, EW, TY, TAS, and HIW conducted the *in vivo* experiment and revised the manuscript and II conducted the data analysis and revised the manuscript. All authors read and approved the final manuscript.
